# Treatment efficacy of azithromycin 1 g single dose versus doxycycline 100 mg twice daily for 7 days for the treatment of rectal chlamydia among men who have sex with men – a double-blind randomised controlled trial protocol

**DOI:** 10.1186/s12879-016-2125-7

**Published:** 2017-01-06

**Authors:** Andrew Lau, Fabian Kong, Christopher K. Fairley, Basil Donovan, Marcus Chen, Catriona Bradshaw, Mark Boyd, Janaki Amin, Peter Timms, Sepehr Tabrizi, David G. Regan, David A. Lewis, Anna McNulty, Jane S. Hocking

**Affiliations:** 1 Centre for Epidemiology and Biostatistics, Melbourne School of Population and Global Health, University of Melbourne, Carlton, 3053 VIC Australia; 2 Melbourne Sexual Health Centre, Alfred Health, 580 Swanston St, Carlton, 3053 VIC Australia; 3 Central Clinical School, Monash University, Clayton, 3800 VIC Australia; 4 The Kirby Institute, UNSW Australia, Kensington, 2052 NSW Australia; 5 University of the Sunshine Coast, 90 Sippy Downs Drive, Sippy Downs, 4556 QLD Australia; 6 Division of Laboratory Services, Department of Microbiology, University of Melbourne, Carlton, 3053 VIC Australia; 7 Western Sydney Sexual Health Centre, 162 Marsden St, Parramatta, 2150 NSW Australia; 8 Sydney Sexual Health Centre, Level 3 Nightingale Wing, Sydney Hospital, Macquarie St, Sydney, 2000 NSW Australia; 9 Marie Bashir Institute for Infectious Diseases and Biosecurity & Sydney Medical School-Westmead, University of Sydney, Sydney, 2000 NSW Australia

**Keywords:** Chlamydia, rectal, treatment, MSM, azithromycin, doxycycline

## Abstract

**Background:**

Rectal infection with *Chlamydia trachomatis* is one of the most common bacterial sexually transmissible infections among men who have sex with men (MSM) with diagnosis rates continuing to rise. Current treatment guidelines recommend either azithromycin 1 g single dose or doxycycline 100 mg twice daily for 7 days. However, there are increasing concerns about treatment failure with azithromycin. We are conducting the first randomised controlled trial (RCT) to compare treatment efficacy of azithromycin versus doxycycline for the treatment of rectal chlamydia in MSM.

**Methods/Design:**

The Rectal Treatment Study will recruit 700 MSM attending Australian sexual health clinics for the treatment of rectal chlamydia. Participants will be asked to provide rectal swabs and will be randomised to either azithromycin 1 g single dose or doxycycline 100 mg twice daily for 7 days. Participants will be asked to complete questionnaires about adverse drug reactions, sexual behaviour and drug adherence via short message service and online survey. The primary outcome is the treatment efficacy as determined by a negative chlamydia nucleic acid amplification test at 4 weeks post treatment. Secondary outcomes will utilise whole genome sequencing and mRNA assay to differentiate between treatment failure, reinfection or false positive results.

**Discussion:**

Rectal chlamydia is an increasing public health concern as use of pre-exposure prophylaxis against HIV becomes commonplace. Optimal, evidence-based treatment is critical to halting ongoing transmission. This study will provide the first RCT evidence comparing azithromycin and doxycycline for the treatment of rectal chlamydia. The results of this trial will establish which treatment is more efficacious and inform international management guidelines.

**Trial registration:**

Australian New Zealand Clinical Trials Registry ACTRN12614001125617.

## Background

Anogenital *Chlamydia trachomatis* is the most commonly diagnosed bacterial sexually transmissible infection (STI) with an estimated 131 million new cases of among 15–49 year-olds worldwide in 2012 [1]. In countries that screen both men and women for chlamydia, men account for approximately 40% of all new diagnoses [2–5]. Although these data do not differentiate between urethral, pharyngeal or rectal infection, available prevalence data among men who have sex with men (MSM) report that rectal infection (5.6–8.2%) is more common than urethral infection (2.1–5.4%) [6–9], with the L2b serovariant indicated in Lymphogranuloma venereum (LGV) [10].

The widespread uptake of biomedical prevention such as pre-exposure prophylaxis (PrEP) for HIV is likely to lead to further increases in rectal chlamydia among MSM [11]. Data from PrEP implementation studies in the US and Australia are showing an increased incidence of rectal STIs among MSM using PrEP [12–14]. The NSW PrEP Demonstration Project found an annual incidence of rectal chlamydia of 67.5% [14] with rates of between 33 and 48% observed in similar projects in the US [12, 13].

Current STI treatment guidelines recommend either azithromycin 1 g single dose or doxycycline 100 mg twice daily for 7 days for the treatment of rectal chlamydia [15–18]. However, there has been increasing concern about the effectiveness of azithromycin for rectal chlamydia with observational studies reporting failure rates of up to 22% [19–22].

In response to these concerns, guidelines in several countries are now recommending that rectal chlamydia infections are treated with 7 days doxycycline [15, 16, 18], rather than 1 g azithromycin as first line. However, there are potential problems adhering to a 7-day course of doxycycline which can lead to an increased risk of treatment failure [22].

A recent meta-analysis reported a treatment efficacy of approximately 83% for azithromycin 1 g single dose compared with 99% for doxycycline 100 mg twice daily for 7 days for rectal chlamydia infection [23]. However, this analysis was based on observational data only with no available evidence from randomized control trials (RCTs). Given that STI rates may continue to increase among MSM and that there is increasing concern about rectal chlamydia among women [24–26], treatment for rectal chlamydia must be proven to be efficacious. A double-blind RCT is urgently needed to rigorously estimate the difference in efficacy between azithromycin and doxycycline. The results will inform treatment guidelines worldwide.

### Research aim and hypothesis

The primary aim is to estimate the efficacy of azithromycin 1 g single dose for the treatment of rectal chlamydia among MSM and compare it to that of doxycycline 100 mg, twice daily for 7 days.

We hypothesize that doxycycline (100 mg, twice daily for 7 days) will be superior to azithromycin (1 g single dose) for the treatment of rectal chlamydia.

## Methods/Design

### Study design and setting

This is a double-blind (clinicians and patients) RCT. Given our primary outcome is treatment efficacy, our trial is double blind to minimize bias that could arise as a result of the different dosing regimens of the two drugs (7 days vs single dose). For example: i) it is possible that taking a 7-day course of daily doxycycline rather than a single dose of azithromycin may deter people from resuming sexual activity while taking treatment, thereby reducing their risk of a new infection, and; ii) participants could be less adherent to a 7-day regimen which could impact efficacy [27, 28]. The trial will be conducted within sexual health clinics in Victoria and New South Wales in Australia and in accordance with the Declaration of Helsinki. The trial was approved by the Alfred Hospital Ethics Committee (373/15).

*(NOTE: hereafter, “azithromycin” refers to azithromycin 1 g single dose and “doxycycline” refers to doxycycline (100 mg, twice daily for 7 days).*

### Duration of study

The trial will be of four weeks duration for each participant.

### Participant eligibility

#### Inclusion criteria

Men will be eligible for inclusion if they report male to male sexual contact in the past 12 months, are aged ≥16 years and test positive for rectal chlamydia using a nucleic acid amplification test (NAAT). They must have adequate English and comprehension skills to give informed consent.

HIV positive MSM will be eligible to participate as there is no evidence that treatment efficacy differs by HIV status. A recent RCT comparing azithromycin with doxycycline for the treatment of chlamydia urethritis found no difference in efficacy between HIV positive and negative men [29]. International STI management guidelines do not differentiate by HIV status for chlamydia treatment [15–18, 30].

#### Exclusion criteria

Men will be excluded if they: i) report use of antibiotics for other purposes in the last 2 weeks; ii) have a known contraindication to the use of azithromycin or doxycycline including allergy; iii) present with symptomatic proctitis. Because MSM will be recruited at clinical services, it will not be possible to genotype their infections to detect LGV at recruitment. LGV genotyping will take place at the conclusion of the trial. Men who are randomised and subsequently found to have asymptomatic LGV based on genotyping at the conclusion of the trial, estimated to be <6% of the trial population [31] will be excluded from our analysis as LGV requires more prolonged doxycycline treatment for cure [32]. The sample size will account for this – see *Sample Size*.

### Recruitment

**Fig. 1:**
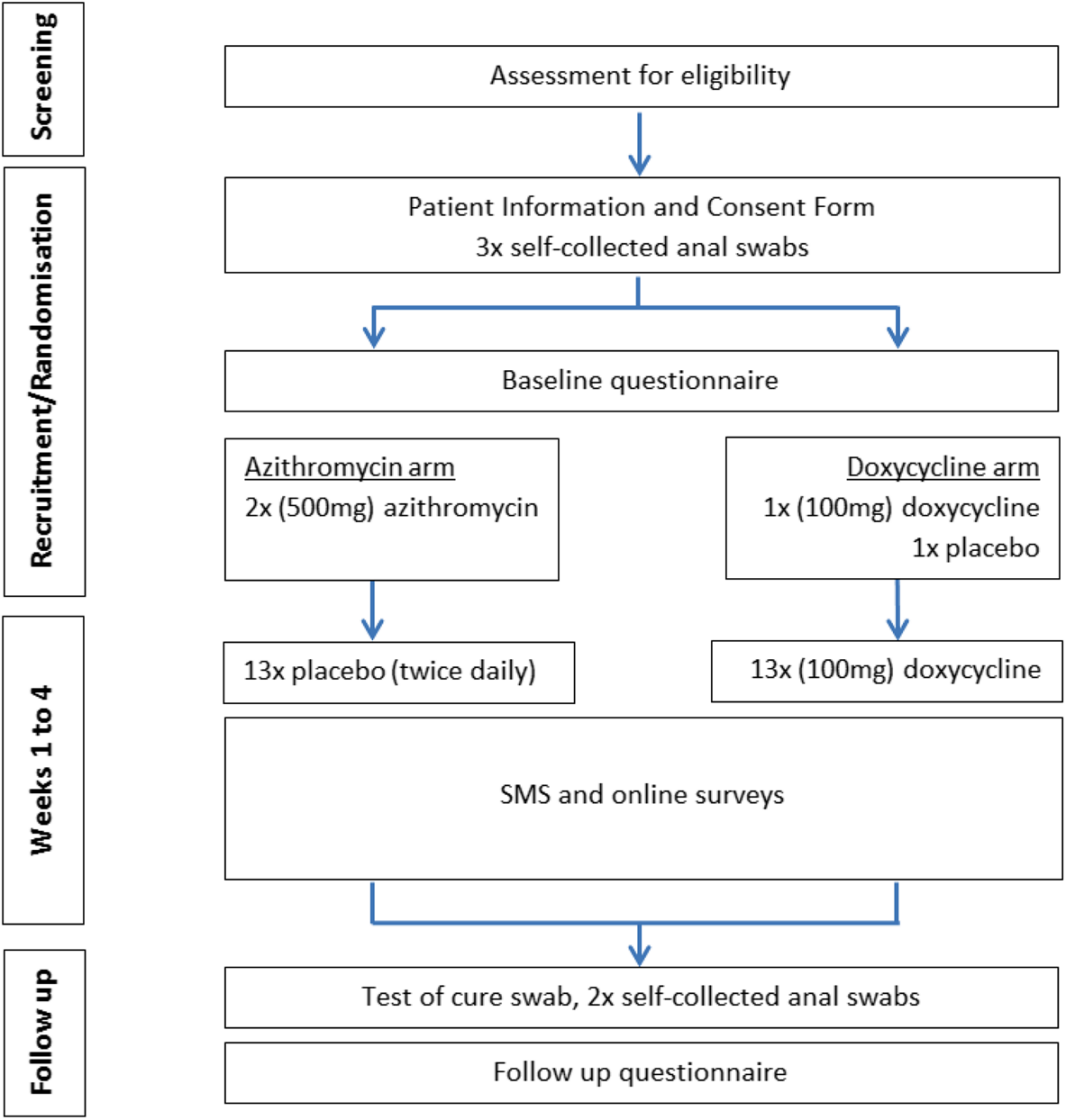
Outline of trial schema. Follow up at 4 weeks post recruitment

MSM who are diagnosed with asymptomatic rectal chlamydia at participating clinics will be approached by a research nurse and invited to take part in the trial (Fig. 1). The nurse will explain the trial, assess eligibility and obtain consent. Eligible men will be enrolled and randomly assigned to either azithromycin or doxycycline. Participants will provide three self-collected rectal swab samples and complete a brief questionnaire. Self-collected swabs have been shown to have similar test performance compared with clinician-collected swabs for chlamydia testing [33]. Once randomly allocated to a treatment group, the nurse will then directly observe participants taking the first dose of treatment (with food).

### Intervention

Participants will be randomly allocated to one of the following treatment groups:

Azithromycin: Participants will receive 1 g of active azithromycin (500 mg tablets × 2), plus 13 placebo doxycycline tablets, identical in appearance to the active azithromycin. Participants will be required to take the two azithromycin tablets under observation at time of recruitment and then advised to take a single placebo tablet morning and night for the next 7 days;

Doxycycline: Participants will receive 14 active doxycycline tablets (100 mg), plus one placebo azithromycin tablet, all identical in appearance to active azithromycin. Participants will be required to take one active doxycycline and one placebo azithromycin tablets under observation at time of recruitment and then advised to take a single active doxycycline tablet morning and night for the next 7 days.

Participants will be advised to take the drugs with food to minimize gastrointestinal side effects and to minimize sun exposure or use sun screen to reduce the risk of photosensitivity.

### Randomization and sequence generation, allocation concealment and blinding

A computer-generated randomization sequence will be created by an independent statistician. Blinded therapy will be prepared by an independent organization and labelled with individual kit numbers according to randomization. Study drugs will be packaged into individually numbered kits stored by independent site pharmacists. All tablets will be identical in appearance and feel, and all medications will be packaged identically to maintain blinding. Participants, physicians, nurses, trial statistician and all other trial staff will be masked to treatment group. The effectiveness of blinding will be tested at completion of the trial when participants will be asked to indicate which treatment they thought they received (including being able to indicate “don’t know”).

The side-effect profiles of the drugs will have negligible impact on blinding. They have been widely used for chlamydia for decades at the dosages we will be using. Their side-effect profiles are well established and similar including minor gastrointestinal upset (nausea, stomach cramps, diarrhoea, vomiting, gastric reflux) [34]. Photosensitivity may occur for doxycycline but is more common with longer or higher dosages [35]. Rash is a rare side effect for each drug, occurring in 0.1–1% of cases [34]. Our packaging will clearly state sunscreen should be used and exposure to sun minimalized, thereby reducing the risk of photosensitivity. We examined the side-effect data from treatment trials for urethral/cervical chlamydia and found that among 17 trials, there was no difference in side-effects (24.0% for azithromycin vs 23.0% for doxycycline, p = 0.45) [36].

### Outcomes

#### Primary outcome

Treatment efficacy measured as microbial cure defined as a negative chlamydia NAAT test result performed on a self-collected rectal swab at week 4.

#### Secondary outcomes

**Fig. 2:**
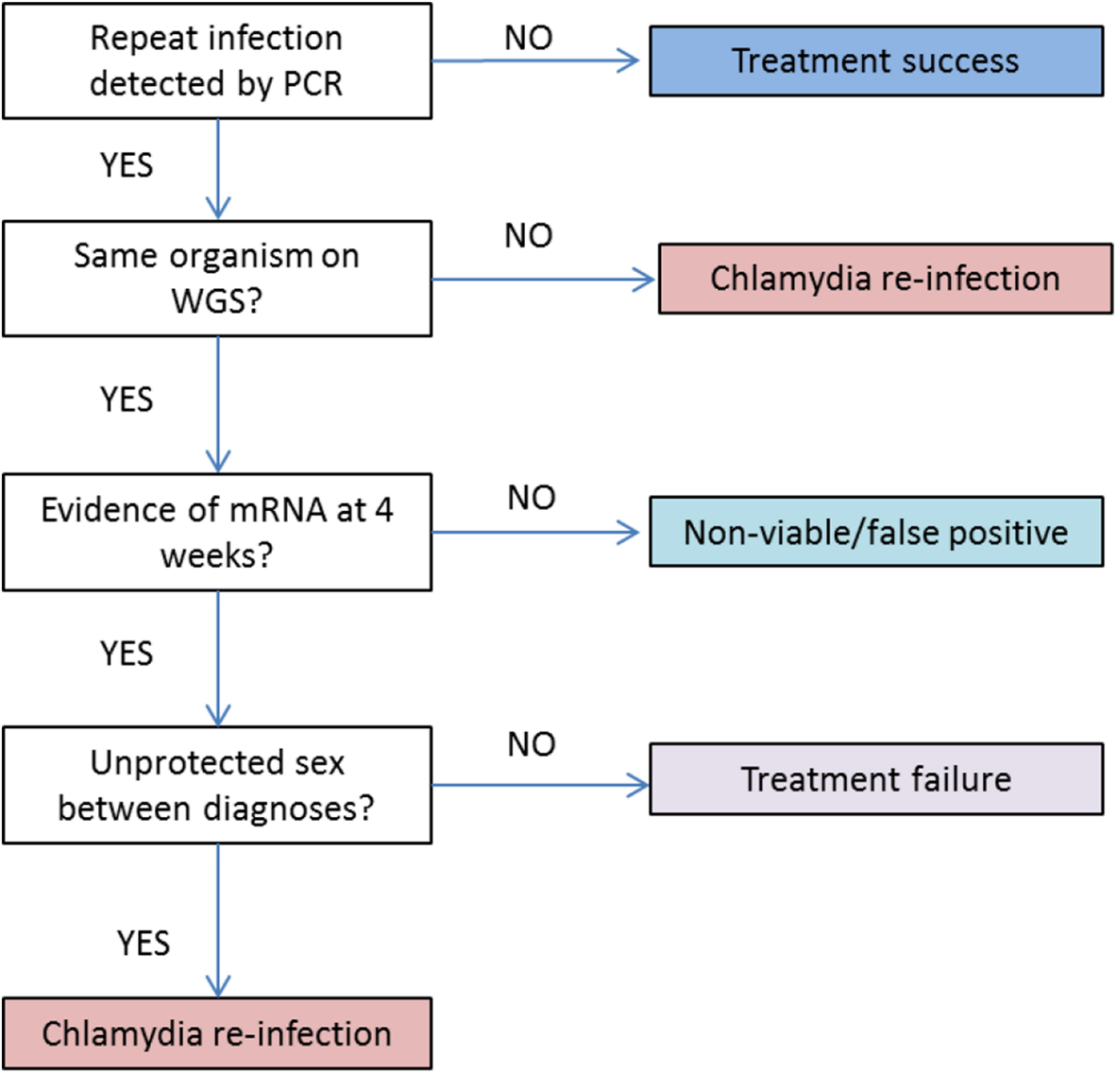
Algorithm for defining repeat infection. PCR = polymerase chain reaction, WGS = whole genome sequencing, mRNA = messenger ribonucleic acid

We will further differentiate between treatment failure and chlamydia re-infection using whole genome sequencing (WGS) and mRNA tests (see below for further detail) for any cases testing chlamydia NAAT positive on a rectal swab at week 4. Using the algorithm (Fig. 2), our secondary outcomes will be classified as: i) microbial cure based on a negative chlamydia NAAT test result at week 4; ii) false positive diagnosis if chlamydia NAAT positive at week 4, but no evidence of mRNA detected [37]; iii) new infection if infected with a different organism (based on sequencing results) OR if the same organism and the participant reports unprotected anal receptive sex between tests; iv) chlamydia treatment failure if infected with exactly the same organism and no reports of unprotected sex between recruitment and week 4 follow up. [NOTE: This algorithm assumes that if a man has a repeat infection with exactly the same organism confirmed by sequencing and reports unprotected sex, he will be classified as having a new infection rather than treatment failure. This is because unprotected sex is one of the greatest risk factors for chlamydia [38]. We acknowledge that this classification will not be 100% accurate, but this level of discrimination has never been previously undertaken in any study of chlamydia treatment efficacy. Our RCT design should ensure that cases classified as a new infection or a false positive diagnosis will be evenly distributed between trial arms, minimising any differential measurement bias.

### Follow up

Men will be required to provide specimens and behavioral data until the conclusion of the trial at 4 weeks (Table 1). Men will be asked to attend the clinic at 4 weeks for a final study visit. At the 4-week visit, the research nurse will administer a final paper questionnaire and additional rectal swabs will be self-collected for WGS and mRNA assay.

**Table 1:** Trial timeline

		Recruitment Day 1	Days 2-6	Day 7	Day 14	Day 21	Follow up Day 28
Recruitment							
Pre-eligibility assessment		X					
Informed consent obtained		X					
Enrollment and randomisation		X					
Dosage under supervision		X					
Data collection	Format						
Baseline questionnaire	Paper	X					
Adverse drug reactions	SMS/Online		X				
Sexual behaviour	SMS/Online			X	X	X	
Other medications	SMS/Online			X	X	X	
Drug adherence	SMS/Online			X			
Follow up questionnaire	Paper						X
Specimen collection	Number						
Self-collected swabs	3	X					
Self-collected swabs	2						X
Test of cure swab	1						X

*SMS* short message service. 3 swabs collected at recuitment: 1 for whole genome sequencing if required, one for mRNA and 1 for culture. 3 swabs collected at follow up: 1 for whole genome sequencing if required, 1 for mRNA and one for test of cure

### Specimen collection

MSM will be asked to provide self-collected rectal swabs.

#### Specimen processing and extraction

All swabs will be sent to the Molecular Microbiology Laboratory, Royal Women’s Hospital for processing and testing. Swabs collected for routine NAAT and WGS will be rotated in 400 μL phosphate buffered saline for 30 s. Swabs collected for mRNA will be rotated in 1 ml of RNAlater (Lifetechnologies) preservative solution (Ambion, Austin, TX, USA) and stored at -80 °C until further testing is required. An aliquot of 200 μl will be extracted by the automated MagNA Pure 96 isolation and purification system (Roche Diagnostics, Mannheim, Germany) using the DNA and Viral NA Small Volume isolation kit. Following nucleic acid isolation, all samples will be initially assessed for DNA and RNA adequacy with a quantitative PCR for a 260 bp fragment of the human beta-globin gene [39] and 226 bp fragment of U1A transcript. [40] Chlamydia testing will be done by the Cobas 4800 CT/NG assay (Roche Applied Science) as per the manufacturer’s instructions.

#### Genotype (strain) determination

Identification of each chlamydia strain including LGV will be determined by qPCR assays using serovar-specific probes as we have described previously [41].

#### Whole genome sequencing (WGS)

Samples from MSM who test chlamydia NAAT positive at week 4 with the same genotype as their baseline sample will undergo WGS to identify the specific strain for each specimen and identify whether the organisms are identical. We will use a direct DNA probe capture method to capture chlamydial DNA directly from the swab sample and sequence the entire genome [42, 43]. Others have used OmpA genotyping or multi-locus sequence typing to characterise genovar [44], but these techniques are considerably less discriminatory than WGS. Our trial will be the first to use WGS to help differentiate between new infection and treatment failure.

#### mRNA

To ensure only actively transcribed nucleic acid is evaluated (as a marker of active, viable infection), extracted nucleic acid will be treated with 10U/μl DNase (Roche Diagnostic) for 10 min at 37 °C, followed by inactivation of DNase. Resultant RNA will undergo one-step reverse transcription and qPCR using the method by Storm et al. [37]. Any case that has mRNA detected will be classified as a true positive. Cases in which mRNA is not detected will be classified as false positive cases. Our trial will be the first to use mRNA assays to identify false positive cases.

### Data collection (Table 1)

#### Questionnaire at recruitment

Participants will complete a paper-based survey at recruitment covering demographics and sexual health, including symptoms and sexual behaviour data. This will include: number of partners; types of sexual activity (including insertive/receptive anal sex); use of intra-rectal devices (eg sex toys); condom use; type of lubrication used and details about douching pre/post sex and types of fluids used for douching. The questions on douching, water based lubricants and intra-rectal devices have been included because they may reduce the antibiotic concentrations in the rectal mucosa due to epithelial damage [45–48]. HIV status, use of pre-exposure prophylaxis for HIV or anti-retroviral therapy, and most recent viral load will be obtained from their medical record.

#### Weekly data collection

Participants will be asked to respond to a weekly short message service (SMS) that collects (via online link) whether they had: any receptive anal sex in the last week; sex with any new sexual partners; sex without a condom in the last week; used any douching and/or intra-rectal devices; any anogenital symptoms; any chlamydia testing elsewhere; taken any further antibiotics.

#### Side effect reporting

During the first week, participants will also be asked daily via SMS whether they had any diarrhoea or vomiting that could impact on their levels of antibiotic absorption.

#### At the 4-week follow up

Participants will complete another paper-based questionnaire that collects information regarding symptoms and sexual behaviour data, diagnosis of any STIs during follow up (which will be validated against their medical record) and what treatment they believe they received. We will also collect information about any recent HIV viral loads and/or CD4 counts.

### Drug adherence monitoring

Participants will be asked to complete a questionnaire about drug adherence at the end of week 1. They will also be asked to return the pill bottle for a pill count as proxy measure of drug adherence. A previous study comparing self-report with measured adherence using the Medication Event Monitoring System, found 83% concordance between self-report and measured adherence for taking 11–14 doses among 206 men and women [28].

### Adverse events reporting

We do not expect any severe adverse events as these drugs have been widely used for decades and their side-effect profiles are well-established. Nevertheless, we will record adverse events which will be coded using the Medical Dictionary for Regulatory Activities (MedDRA). The percentage of patients with treatment-emergent adverse events will be tabulated by system organ class and severity of these events with particular focus on gastrointestinal events together with severity of these events [49].

### Sample size

Our hypothesis is that azithromycin efficacy will be less than that of doxycycline. On the basis of our meta-analysis, we assume that the microbial cure among the doxycycline arm at 4 weeks will be 98% compared to azithromycin at 93%. We will recruit 700 men in total (350 in each group) which, allowing for a very conservative 14% loss to follow up (based on our experience with similar trials of MSM) [50] and a further loss of 6% due to a LGV diagnosis at the end of the trial, will give us an effective sample size of 560. This sample size will allow us to detect a 5% difference between doxycycline and azithromycin microbial cure at 4 weeks with 80% power and a 6% difference with 90% power. If microbial cure is 96% among those treated with doxycycline at week 4, then we will have 80% power to detect a 6% difference and 90% power to detect a 7% difference (Table 2).

**Table 2:** Sample size calculation

Doxycycline cure	98% cure		96% cure	
Power	80%	90%	80%	90%
Difference to detect^a^				
%	538	720	762	1018
6%	412	552	560	756
7%	332	442	444	592

^a^Assumes azithromycin microbial cure will be 5–7% lower. Sample size calculation assumes 5% significance

### Analysis

We will compare the proportion with microbial cure at 4 weeks between arms using the Chi square test, and 95% confidence intervals for the difference between proportions reported. While randomisation should ensure balance of baseline characteristics, if there are meaningful differences in baseline characteristics including sexual behaviour, a multivariable logistic regression analysis will be undertaken to adjust for these factors.

#### Primary analysis

Our primary analysis will be a modified intention to treat analysis (m-ITT) including only those who commenced randomised therapy at recruitment (at least one dose) and those who provided a rectal specimen for chlamydia testing at week 4.

#### Secondary analysis

We will undertake two per protocol analyses in which participants who took less than 10 doses of their allocated treatment [28] or vomited within 1 h of any dose will be excluded: i) an analysis of the primary outcome of the microbial cure, and; ii) an analysis of the secondary outcome of microbial cure in which cases classified as new infection or a false diagnosis are grouped with microbial cure to create a binary variable for the analysis (microbial cure [including new infection and false positive cases] vs treatment failure).

### Trial status

The trial has been registered on the Australian and New Zealand Clinical Trials Registry (ACTRN12614001125617). The trial commenced recruitment in August 2016 and is due to be completed by August 2019.

## Discussion

Given rectal chlamydia is highly prevalent among MSM and is likely to increase, it is vital that the most efficacious treatment is used. Currently azithromycin, which meta-analysis suggests might fail in 17% of cases [23], is continuing to be widely used. This will be the first RCT to compare azithromycin and doxycycline for rectal chlamydia and will establish whether doxycycline is more efficacious than azithromycin.

Currently, there are no pharmacokinetic data available for the action of azithromycin in rectal mucosa and a complex interplay of pharmacological and immunological factors means that azithromycin may be less effective for rectal compared with urethral/cervical infections [51]. Azithromycin is delivered to the site of infection by phagocytic cells released during the immune response to infection [52] whereas doxycycline is highly lipid soluble and rapidly absorbed into the tissues [53]. Data from both human studies [54] and mice models [55] suggest that chlamydia down-regulates the immune response in the gastrointestinal tract, and may therefore reduce the number of phagocytic cells available to deliver the azithromycin. Other mouse studies have shown that gastrointestinal chlamydiae are less susceptible to clearance by azithromycin than genital species [55]. Therefore, it is biologically plausible that a reduced local immune response in the rectum may attenuate azithromycin efficacy.

We have limited the trial to MSM because most rectal chlamydia is diagnosed among MSM and regular screening for rectal STIs is recommended only for this population [16]. Nevertheless, our results will have implications for women in whom rectal chlamydia can be diagnosed [56]. The biology of rectal infection is likely to be similar between men and women with similar rectal organism loads reported for both men and women [57]. In addition, Gratrix et al. found no significant difference in rectal treatment efficacy between men and women [58]. As such, we believe our results will be generalizable to women.

A repeat positive chlamydia test at follow up does not necessarily indicate treatment failure; it could be a re-infection or a false positive result. A strength of this trial is that we can take advantage of the latest technology to more accurately discriminate between treatment failure and re-infection or false positive result. We will use WGS to differentiate between new infection and treatment failure. WGS is the most discriminatory tool available as it can identify conclusively whether the specific strain at follow up is different from that at recruitment thereby indicating a new infection. Previously, studies have used O*mpA* genotyping or multi-locus sequence typing (MLST) to characterise the genovar [44], but these techniques are considerably less discriminatory than WGS. We will use mRNA tests to identify false positive cases. Standard chlamydia tests detect chlamydia nucleic acid which includes both viable and non-viable (dead) organisms. When antibiotic treatment kills chlamydia, it can take 3 weeks to clear the nucleic acid from the genital tract [59], so any NAAT conducted during this time may detect non-viable organism only and be a false positive. Chlamydial mRNA assays use a quantitative real-time chlamydia molecular test (see p7) and will quantitatively measure mRNA transcription of omp2 (omcB) outer membrane protein in chlamydia as an expression of viable organisms (active infection) [37]. In addition, we will collect comprehensive sexual practice data to identify men at risk of re-infection using SMS.

This trial must be done to ensure STI management guidelines internationally are evidence-based and recommend the most efficacious treatment for rectal chlamydia so that ongoing transmission is minimised.

## Abbreviations

HIV: Human immuno-deficiency virus
LGV: Lymphogranuloma venereum
mITT: Modified intention to treat
MLST: Multi-locus sequence typing
MSM: Men who have sex with men
NAAT: Nucleic acid amplification test
NSW: New South Wales
PCR: Polymerase chain reaction
PrEP: Pre-exposure prophylaxis
RCT: Randomised controlled trial
SMS: Short message service
STI: Sexually transmissible infection
WGS: Whole genome sequencing
